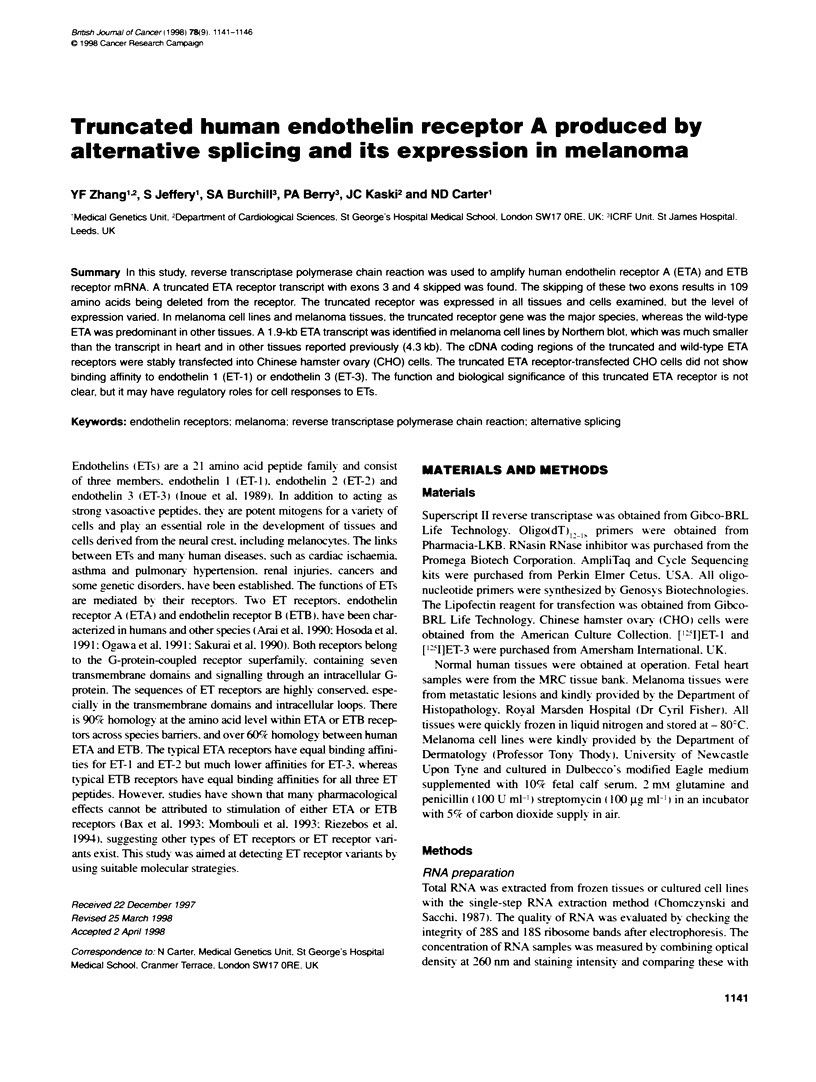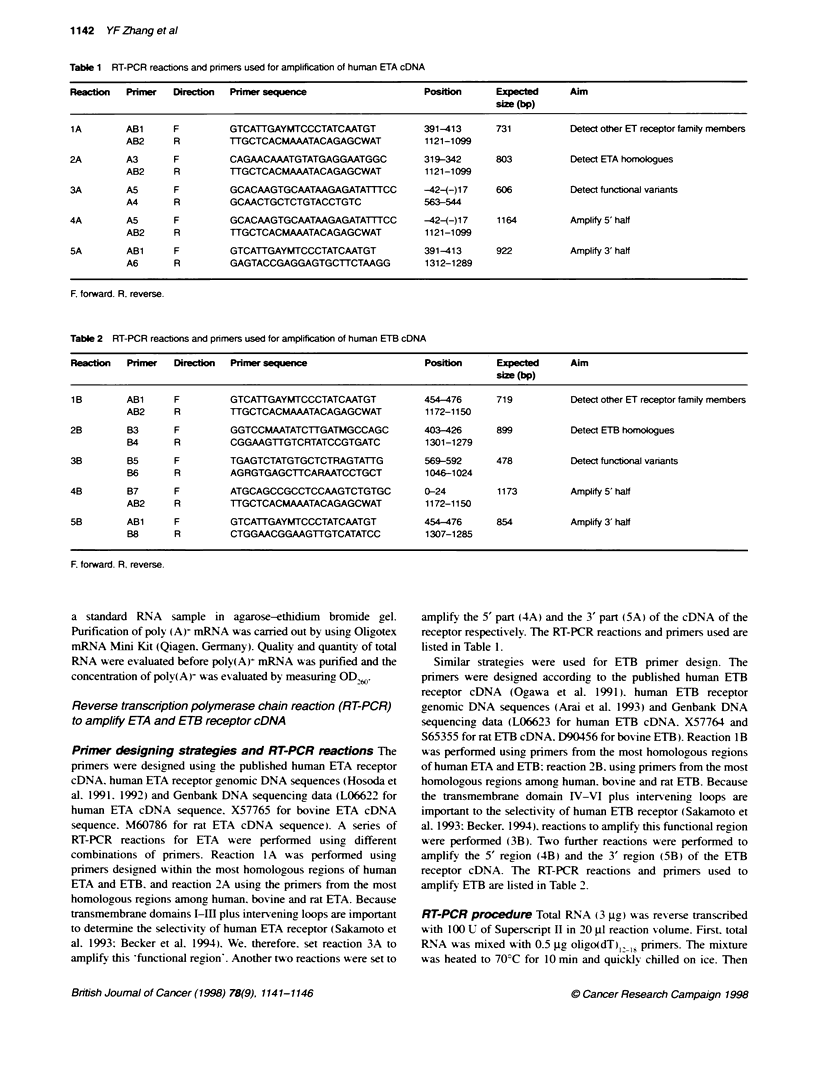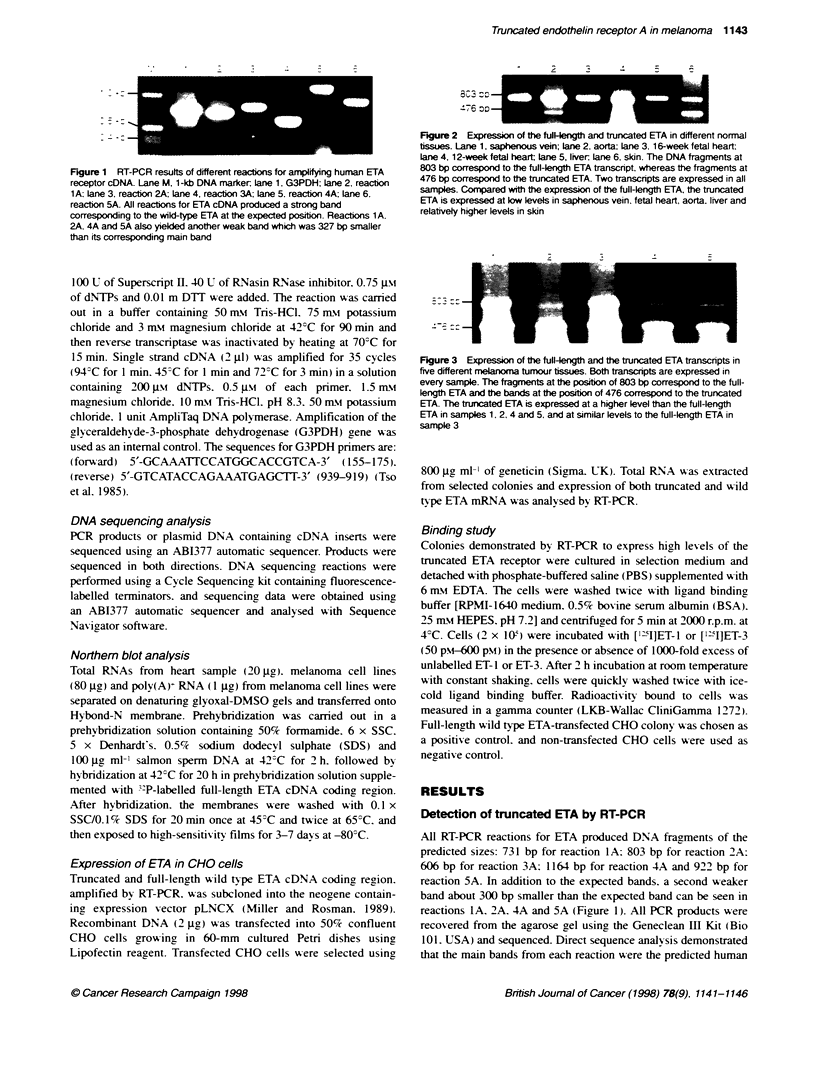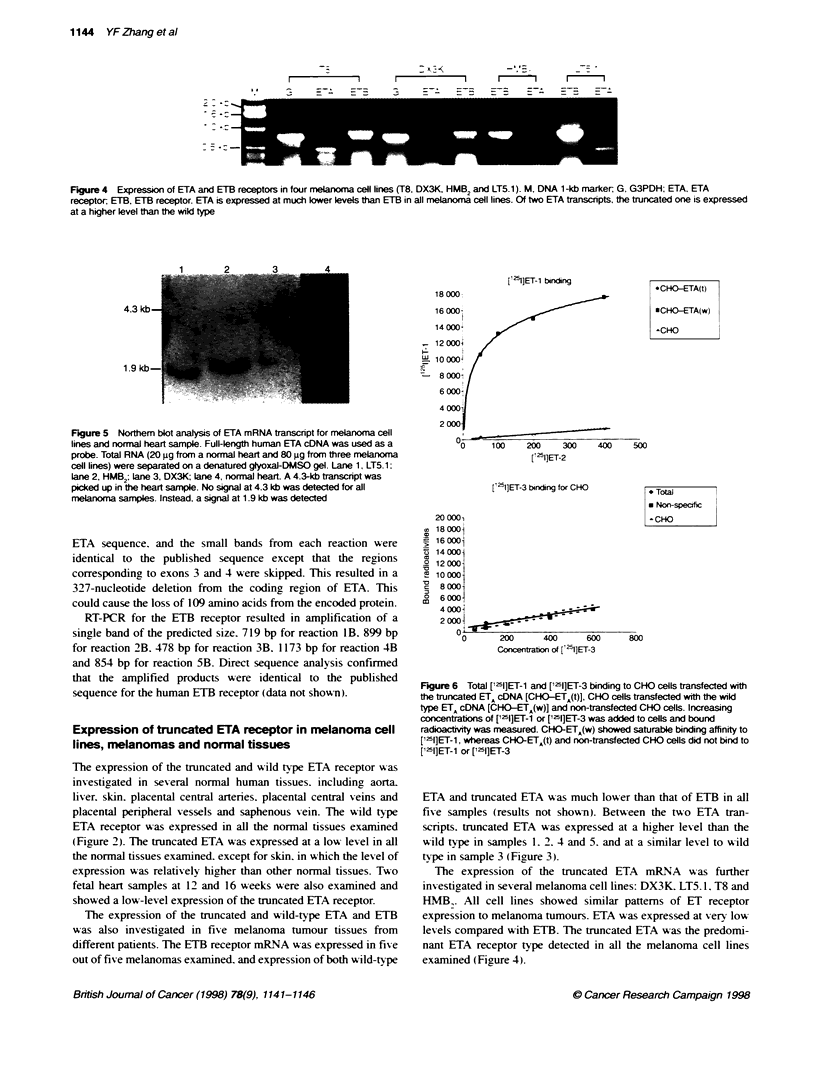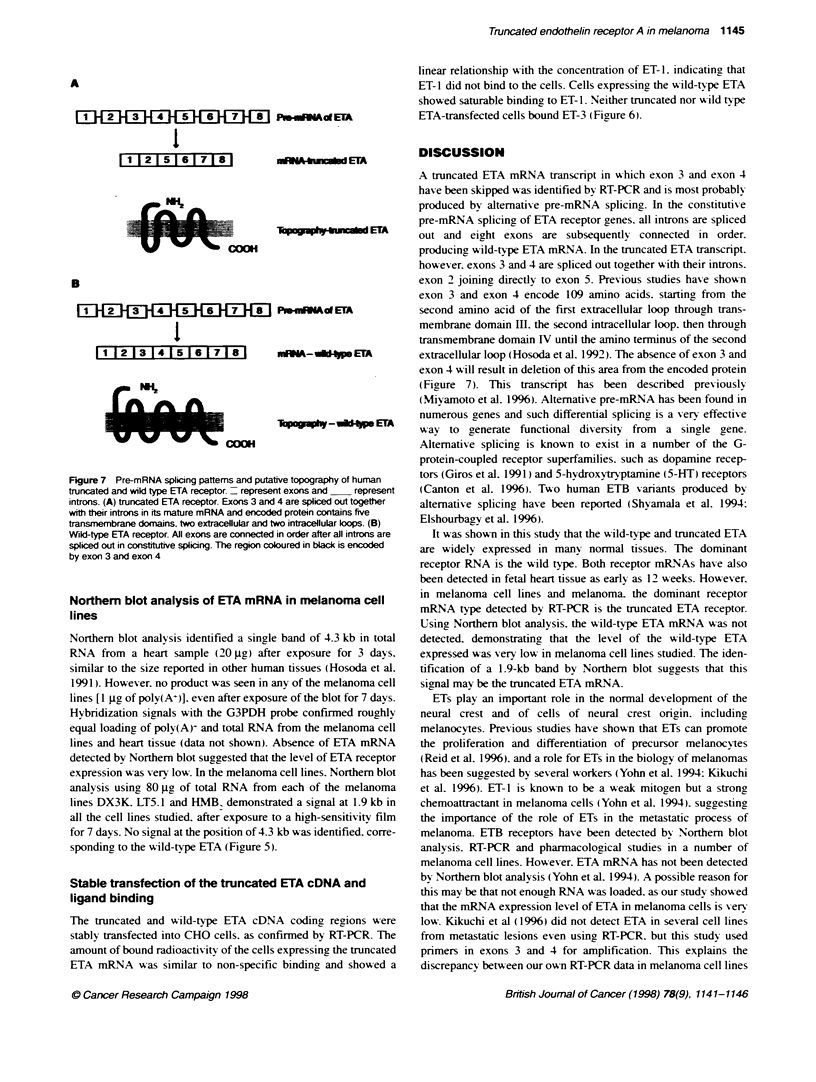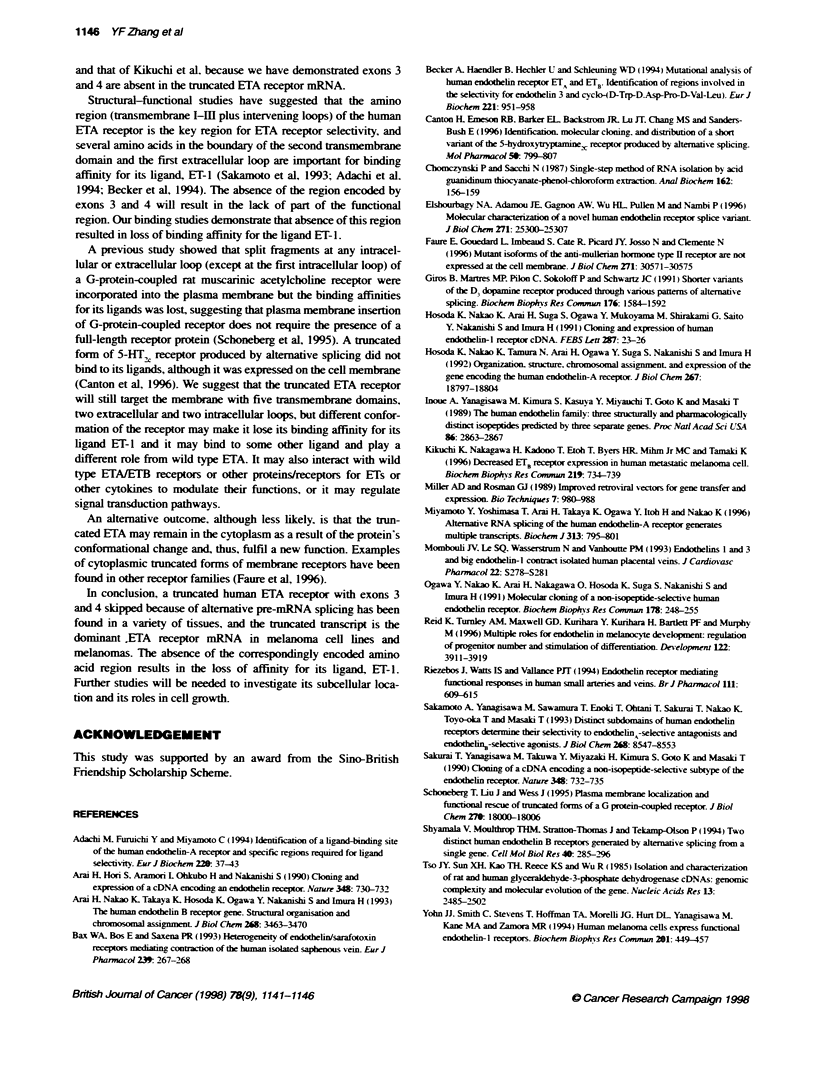# Truncated human endothelin receptor A produced by alternative splicing and its expression in melanoma.

**DOI:** 10.1038/bjc.1998.643

**Published:** 1998-11

**Authors:** Y. F. Zhang, S. Jeffery, S. A. Burchill, P. A. Berry, J. C. Kaski, N. D. Carter

**Affiliations:** Department of Cardiological Sciences, St George's Hospital Medical School, London, UK.

## Abstract

**Images:**


British Journal of Cancer (1998) 78(9), 1141–1146
© 1998 Cancer Research Campaign

### Truncated human endothelin receptor A produced by alternative splicing and its expression in melanoma

**YF Zhang[1,2], S Jeffery[1], SA Burchill[3], PA Berry[3], JC Kaski[2] and ND Carter[1]**

[1]Medical Genetics Unit, [2]Department of Cardiological Sciences, St George's Hospital Medical School, London SW17 0RE, UK; [3]ICRF Unit, St James Hospital, Leeds, UK

**Summary** In this study, reverse transcriptase polymerase chain reaction was used to amplify human endothelin receptor A (ETA) and ETB receptor mRNA. A truncated ETA receptor transcript with exons 3 and 4 skipped was found. The skipping of these two exons results in 109 amino acids being deleted from the receptor. The truncated receptor was expressed in all tissues and cells examined, but the level of expression varied. In melanoma cell lines and melanoma tissues, the truncated receptor gene was the major species, whereas the wild-type ETA was predominant in other tissues. A 1.9-kb ETA transcript was identified in melanoma cell lines by Northern blot, which was much smaller than the transcript in heart and in other tissues reported previously (4.3 kb). The cDNA coding regions of the truncated and wild-type ETA receptors were stably transfected into Chinese hamster ovary (CHO) cells. The truncated ETA receptor-transfected CHO cells did not show binding affinity to endothelin 1 (ET-1) or endothelin 3 (ET-3). The function and biological significance of this truncated ETA receptor is not clear, but it may have regulatory roles for cell responses to ETs.

**Keywords:** endothelin receptors; melanoma; reverse transcriptase polymerase chain reaction; alternative splicing

Endothelins (ETs) are a 21 amino acid peptide family and consist of three members, endothelin 1 (ET-1), endothelin 2 (ET-2) and endothelin 3 (ET-3) (Inoue et al, 1989). In addition to acting as strong vasoactive peptides, they are potent mitogens for a variety of cells and play an essential role in the development of tissues and cells derived from the neural crest, including melanocytes. The links between ETs and many human diseases, such as cardiac ischaemia, asthma and pulmonary hypertension, renal injuries, cancers and some genetic disorders, have been established. The functions of ETs are mediated by their receptors. Two ET receptors, endothelin receptor A (ETA) and endothelin receptor B (ETB), have been characterized in humans and other species (Arai et al, 1990; Hosoda et al, 1991; Ogawa et al, 1991; Sakurai et al, 1990). Both receptors belong to the G-protein-coupled receptor superfamily, containing seven transmembrane domains and signalling through an intracellular G-protein. The sequences of ET receptors are highly conserved, especially in the transmembrane domains and intracellular loops. There is 90% homology at the amino acid level within ETA or ETB receptors across species barriers, and over 60% homology between human ETA and ETB. The typical ETA receptors have equal binding affinities for ET-1 and ET-2 but much lower affinities for ET-3, whereas typical ETB receptors have equal binding affinities for all three ET peptides. However, studies have shown that many pharmacological effects cannot be attributed to stimulation of either ETA or ETB receptors (Bax et al, 1993; Mombouli et al, 1993; Riezebos et al, 1994), suggesting other types of ET receptors or ET receptor variants exist. This study was aimed at detecting ET receptor variants by using suitable molecular strategies.

*Received 22 December 1997*
*Revised 25 March 1998*
*Accepted 2 April 1998*

*Correspondence to:* N Carter, Medical Genetics Unit, St George's Hospital Medical School, Cranmer Terrace, London SW17 0RE, UK

### MATERIALS AND METHODS

### Materials

Superscript II reverse transcriptase was obtained from Gibco-BRL Life Technology. Oligo(dT)$_{12-18}$ primers were obtained from Pharmacia-LKB. RNasin RNase inhibitor was purchased from the Promega Biotech Corporation. AmpliTaq and Cycle Sequencing kits were purchased from Perkin Elmer Cetus, USA. All oligonucleotide primers were synthesized by Genosys Biotechnologies. The Lipofectin reagent for transfection was obtained from Gibco-BRL Life Technology. Chinese hamster ovary (CHO) cells were obtained from the American Culture Collection. [$^{125}$I]ET-1 and [$^{125}$I]ET-3 were purchased from Amersham International, UK.

Normal human tissues were obtained at operation. Fetal heart samples were from the MRC tissue bank. Melanoma tissues were from metastatic lesions and kindly provided by the Department of Histopathology, Royal Marsden Hospital (Dr Cyril Fisher). All tissues were quickly frozen in liquid nitrogen and stored at – 80°C. Melanoma cell lines were kindly provided by the Department of Dermatology (Professor Tony Thody), University of Newcastle Upon Tyne and cultured in Dulbecco's modified Eagle medium supplemented with 10% fetal calf serum, 2 mM glutamine and penicillin (100 U ml$^{-1}$) streptomycin (100 μg ml$^{-1}$) in an incubator with 5% of carbon dioxide supply in air.

### Methods

#### *RNA preparation*

Total RNA was extracted from frozen tissues or cultured cell lines with the single-step RNA extraction method (Chomczynski and Sacchi, 1987). The quality of RNA was evaluated by checking the integrity of 28S and 18S ribosome bands after electrophoresis. The concentration of RNA samples was measured by combining optical density at 260 nm and staining intensity and comparing these with

**Table 1** RT-PCR reactions and primers used for amplification of human ETA cDNA

| Reaction | Primer | Direction | Primer sequence | Position | Expected size (bp) | Aim |
|---|---|---|---|---|---|---|
| 1A | AB1 | F | GTCATTGAYMTCCCTATCAATGT | 391–413 | 731 | Detect other ET receptor family members |
| | AB2 | R | TTGCTCACMAAATACAGAGCWAT | 1121–1099 | | |
| 2A | A3 | F | CAGAACAAATGTATGAGGAATGGC | 319–342 | 803 | Detect ETA homologues |
| | AB2 | R | TTGCTCACMAAATACAGAGCWAT | 1121–1099 | | |
| 3A | A5 | F | GCACAAGTGCAATAAGAGATATTTCC | −42–(−)17 | 606 | Detect functional variants |
| | A4 | R | GCAACTGCTCTGTACCTGTC | 563–544 | | |
| 4A | A5 | F | GCACAAGTGCAATAAGAGATATTTCC | −42–(−)17 | 1164 | Amplify 5′ half |
| | AB2 | R | TTGCTCACMAAATACAGAGCWAT | 1121–1099 | | |
| 5A | AB1 | F | GTCATTGAYMTCCCTATCAATGT | 391–413 | 922 | Amplify 3′ half |
| | A6 | R | GAGTACCGAGGAGTGCTTCTAAGG | 1312–1289 | | |

F, forward. R, reverse.

**Table 2** RT-PCR reactions and primers used for amplification of human ETB cDNA

| Reaction | Primer | Direction | Primer sequence | Position | Expected size (bp) | Aim |
|---|---|---|---|---|---|---|
| 1B | AB1 | F | GTCATTGAYMTCCCTATCAATGT | 454–476 | 719 | Detect other ET receptor family members |
| | AB2 | R | TTGCTCACMAAATACAGAGCWAT | 1172–1150 | | |
| 2B | B3 | F | GGTCCMAATATCTTGATMGCCAGC | 403–426 | 899 | Detect ETB homologues |
| | B4 | R | CGGAAGTTGTCRTATCCGTGATC | 1301–1279 | | |
| 3B | B5 | F | TGAGTCTATGTGCTCTRAGTATTG | 569–592 | 478 | Detect functional variants |
| | B6 | R | AGRGTGAGCTTCARAATCCTGCT | 1046–1024 | | |
| 4B | B7 | F | ATGCAGCCGCCTCCAAGTCTGTGC | 0–24 | 1173 | Amplify 5′ half |
| | AB2 | R | TTGCTCACMAAATACAGAGCWAT | 1172–1150 | | |
| 5B | AB1 | F | GTCATTGAYMTCCCTATCAATGT | 454–476 | 854 | Amplify 3′ half |
| | B8 | R | CTGGAACGGAAGTTGTCATATCC | 1307–1285 | | |

F, forward. R, reverse.

a standard RNA sample in agarose–ethidium bromide gel. Purification of poly (A)$^+$ mRNA was carried out by using Oligotex mRNA Mini Kit (Qiagen, Germany). Quality and quantity of total RNA were evaluated before poly(A)$^+$ mRNA was purified and the concentration of poly(A)$^+$ was evaluated by measuring $OD_{260}$.

### *Reverse transcription polymerase chain reaction (RT-PCR) to amplify ETA and ETB receptor cDNA*

***Primer designing strategies and RT-PCR reactions*** The primers were designed using the published human ETA receptor cDNA, human ETA receptor genomic DNA sequences (Hosoda et al, 1991, 1992) and Genbank DNA sequencing data (L06622 for human ETA cDNA sequence, X57765 for bovine ETA cDNA sequence, M60786 for rat ETA cDNA sequence). A series of RT-PCR reactions for ETA were performed using different combinations of primers. Reaction 1A was performed using primers designed within the most homologous regions of human ETA and ETB, and reaction 2A using the primers from the most homologous regions among human, bovine and rat ETA. Because transmembrane domains I–III plus intervening loops are important to determine the selectivity of human ETA receptor (Sakamoto et al, 1993; Becker et al, 1994), We, therefore, set reaction 3A to amplify this 'functional region'. Another two reactions were set to amplify the 5′ part (4A) and the 3′ part (5A) of the cDNA of the receptor respectively. The RT-PCR reactions and primers used are listed in Table 1.

Similar strategies were used for ETB primer design. The primers were designed according to the published human ETB receptor cDNA (Ogawa et al, 1991), human ETB receptor genomic DNA sequences (Arai et al, 1993) and Genbank DNA sequencing data (L06623 for human ETB cDNA, X57764 and S65355 for rat ETB cDNA, D90456 for bovine ETB). Reaction 1B was performed using primers from the most homologous regions of human ETA and ETB; reaction 2B, using primers from the most homologous regions among human, bovine and rat ETB. Because the transmembrane domain IV–VI plus intervening loops are important to the selectivity of human ETB receptor (Sakamoto et al, 1993; Becker, 1994), reactions to amplify this functional region were performed (3B). Two further reactions were performed to amplify the 5′ region (4B) and the 3′ region (5B) of the ETB receptor cDNA. The RT-PCR reactions and primers used to amplify ETB are listed in Table 2.

***RT-PCR procedure*** Total RNA (3 μg) was reverse transcribed with 100 U of Superscript II in 20 μl reaction volume. First, total RNA was mixed with 0.5 μg oligo(dT)$_{12-18}$ primers. The mixture was heated to 70°C for 10 min and quickly chilled on ice. Then

 © Cancer Research Campaign 1998

**Figure 1** RT-PCR results of different reactions for amplifying human ETA receptor cDNA. Lane M, 1-kb DNA marker; lane 1, G3PDH; lane 2, reaction 1A; lane 3, reaction 2A; lane 4, reaction 3A; lane 5, reaction 4A; lane 6, reaction 5A. All reactions for ETA cDNA produced a strong band corresponding to the wild-type ETA at the expected position. Reactions 1A, 2A, 4A and 5A also yielded another weak band which was 327 bp smaller than its corresponding main band

**Figure 2** Expression of the full-length and truncated ETA in different normal tissues. Lane 1, saphenous vein; lane 2, aorta; lane 3, 16-week fetal heart; lane 4, 12-week fetal heart; lane 5, liver; lane 6, skin. The DNA fragments at 803 bp correspond to the full-length ETA transcript, whereas the fragments at 476 bp correspond to the truncated ETA. Two transcripts are expressed in all samples. Compared with the expression of the full-length ETA, the truncated ETA is expressed at low levels in saphenous vein, fetal heart, aorta, liver and relatively higher levels in skin

**Figure 3** Expression of the full-length and the truncated ETA transcripts in five different melanoma tumour tissues. Both transcripts are expressed in every sample. The fragments at the position of 803 bp correspond to the full-length ETA and the bands at the position of 476 correspond to the truncated ETA. The truncated ETA is expressed at a higher level than the full-length ETA in samples 1, 2, 4 and 5, and at similar levels to the full-length ETA in sample 3

100 U of Superscript II, 40 U of RNasin RNase inhibitor, 0.75 μM of dNTPs and 0.01 m DTT were added. The reaction was carried out in a buffer containing 50 mM Tris-HCl, 75 mM potassium chloride and 3 mM magnesium chloride at 42°C for 90 min and then reverse transcriptase was inactivated by heating at 70°C for 15 min. Single strand cDNA (2 μl) was amplified for 35 cycles (94°C for 1 min, 45°C for 1 min and 72°C for 3 min) in a solution containing 200 μM dNTPs, 0.5 μM of each primer, 1.5 mM magnesium chloride, 10 mM Tris-HCl, pH 8.3, 50 mM potassium chloride, 1 unit AmpliTaq DNA polymerase. Amplification of the glyceraldehyde-3-phosphate dehydrogenase (G3PDH) gene was used as an internal control. The sequences for G3PDH primers are: (forward) 5′-GCAAATTCCATGGCACCGTCA-3′ (155–175), (reverse) 5′-GTCATACCAGAAATGAGCTT-3′ (939–919) (Tso et al, 1985).

### DNA sequencing analysis

PCR products or plasmid DNA containing cDNA inserts were sequenced using an ABI377 automatic sequencer. Products were sequenced in both directions. DNA sequencing reactions were performed using a Cycle Sequencing kit containing fluorescence-labelled terminators, and sequencing data were obtained using an ABI377 automatic sequencer and analysed with Sequence Navigator software.

### Northern blot analysis

Total RNAs from heart sample (20 μg), melanoma cell lines (80 μg) and poly(A)$^+$ RNA (1 μg) from melanoma cell lines were separated on denaturing glyoxal-DMSO gels and transferred onto Hybond-N membrane. Prehybridization was carried out in a prehybridization solution containing 50% formamide, 6 × SSC, 5 × Denhardt's, 0.5% sodium dodecyl sulphate (SDS) and 100 μg ml$^{-1}$ salmon sperm DNA at 42°C for 2 h, followed by hybridization at 42°C for 20 h in prehybridization solution supplemented with $^{32}$P-labelled full-length ETA cDNA coding region. After hybridization, the membranes were washed with 0.1 × SSC/0.1% SDS for 20 min once at 45°C and twice at 65°C, and then exposed to high-sensitivity films for 3–7 days at –80°C.

### Expression of ETA in CHO cells

Truncated and full-length wild type ETA cDNA coding region, amplified by RT-PCR, was subcloned into the neogene containing expression vector pLNCX (Miller and Rosman, 1989). Recombinant DNA (2 μg) was transfected into 50% confluent CHO cells growing in 60-mm cultured Petri dishes using Lipofectin reagent. Transfected CHO cells were selected using 800 μg ml$^{-1}$ of geneticin (Sigma, UK). Total RNA was extracted from selected colonies and expression of both truncated and wild type ETA mRNA was analysed by RT-PCR.

### Binding study

Colonies demonstrated by RT-PCR to express high levels of the truncated ETA receptor were cultured in selection medium and detached with phosphate-buffered saline (PBS) supplemented with 6 mM EDTA. The cells were washed twice with ligand binding buffer [RPMI-1640 medium, 0.5% bovine serum albumin (BSA), 25 mM HEPES, pH 7.2] and centrifuged for 5 min at 2000 r.p.m. at 4°C. Cells (2 × $10^5$) were incubated with [$^{125}$I]ET-1 or [$^{125}$I]ET-3 (50 pM–600 pM) in the presence or absence of 1000-fold excess of unlabelled ET-1 or ET-3. After 2 h incubation at room temperature with constant shaking, cells were quickly washed twice with ice-cold ligand binding buffer. Radioactivity bound to cells was measured in a gamma counter (LKB-Wallac CliniGamma 1272). Full-length wild type ETA-transfected CHO colony was chosen as a positive control, and non-transfected CHO cells were used as negative control.

### RESULTS

### Detection of truncated ETA by RT-PCR

All RT-PCR reactions for ETA produced DNA fragments of the predicted sizes: 731 bp for reaction 1A; 803 bp for reaction 2A; 606 bp for reaction 3A; 1164 bp for reaction 4A and 922 bp for reaction 5A. In addition to the expected bands, a second weaker band about 300 bp smaller than the expected band can be seen in reactions 1A, 2A, 4A and 5A (Figure 1). All PCR products were recovered from the agarose gel using the Geneclean III Kit (Bio 101, USA) and sequenced. Direct sequence analysis demonstrated that the main bands from each reaction were the predicted human

© Cancer Research Campaign 1998 

**Figure 4** Expression of ETA and ETB receptors in four melanoma cell lines (T8, DX3K, $HMB_2$ and LT5.1). M, DNA 1-kb marker; G, G3PDH; ETA, ETA receptor; ETB, ETB receptor. ETA is expressed at much lower levels than ETB in all melanoma cell lines. Of two ETA transcripts, the truncated one is expressed at a higher level than the wild type

**Figure 5** Northern blot analysis of ETA mRNA transcript for melanoma cell lines and normal heart sample. Full-length human ETA cDNA was used as a probe. Total RNA (20 µg from a normal heart and 80 µg from three melanoma cell lines) were separated on a denatured glyoxal-DMSO gel. Lane 1, LT5.1; lane 2, $HMB_2$; lane 3, DX3K; lane 4, normal heart. A 4.3-kb transcript was picked up in the heart sample. No signal at 4.3 kb was detected for all melanoma samples. Instead, a signal at 1.9 kb was detected

ETA sequence, and the small bands from each reaction were identical to the published sequence except that the regions corresponding to exons 3 and 4 were skipped. This resulted in a 327-nucleotide deletion from the coding region of ETA. This could cause the loss of 109 amino acids from the encoded protein.

RT-PCR for the ETB receptor resulted in amplification of a single band of the predicted size, 719 bp for reaction 1B, 899 bp for reaction 2B, 478 bp for reaction 3B, 1173 bp for reaction 4B and 854 bp for reaction 5B. Direct sequence analysis confirmed that the amplified products were identical to the published sequence for the human ETB receptor (data not shown).

### Expression of truncated ETA receptor in melanoma cell lines, melanomas and normal tissues

The expression of the truncated and wild type ETA receptor was investigated in several normal human tissues, including aorta, liver, skin, placental central arteries, placental central veins and placental peripheral vessels and saphenous vein. The wild type ETA receptor was expressed in all the normal tissues examined (Figure 2). The truncated ETA was expressed at a low level in all the normal tissues examined, except for skin, in which the level of expression was relatively higher than other normal tissues. Two fetal heart samples at 12 and 16 weeks were also examined and showed a low-level expression of the truncated ETA receptor.

The expression of the truncated and wild-type ETA and ETB was also investigated in five melanoma tumour tissues from different patients. The ETB receptor mRNA was expressed in five out of five melanomas examined, and expression of both wild-type

**Figure 6** Total [$^{125}$I]ET-1 and [$^{125}$I]ET-3 binding to CHO cells transfected with the truncated $ET_A$ cDNA [CHO–$ET_A$(t)], CHO cells transfected with the wild type $ET_A$ cDNA [CHO–$ET_A$(w)] and non-transfected CHO cells. Increasing concentrations of [$^{125}$I]ET-1 or [$^{125}$I]ET-3 was added to cells and bound radioactivity was measured. CHO-$ET_A$(w) showed saturable binding affinity to [$^{125}$I]ET-1, whereas CHO-$ET_A$(t) and non-transfected CHO cells did not bind to [$^{125}$I]ET-1 or [$^{125}$I]ET-3

ETA and truncated ETA was much lower than that of ETB in all five samples (results not shown). Between the two ETA transcripts, truncated ETA was expressed at a higher level than the wild type in samples 1, 2, 4 and 5, and at a similar level to wild type in sample 3 (Figure 3).

The expression of the truncated ETA mRNA was further investigated in several melanoma cell lines: DX3K, LT5.1, T8 and $HMB_2$. All cell lines showed similar patterns of ET receptor expression to melanoma tumours. ETA was expressed at very low levels compared with ETB. The truncated ETA was the predominant ETA receptor type detected in all the melanoma cell lines examined (Figure 4).

 © Cancer Research Campaign 1998

**Figure 7** Pre-mRNA splicing patterns and putative topography of human truncated and wild type ETA receptor. ☐ represent exons and ____ represent introns. (**A**) truncated ETA receptor. Exons 3 and 4 are spliced out together with their introns in its mature mRNA and encoded protein contains five transmembrane domains, two extracellular and two intracellular loops. (**B**) Wild-type ETA receptor. All exons are connected in order after all introns are spliced out in constitutive splicing. The region coloured in black is encoded by exon 3 and exon 4

### Northern blot analysis of ETA mRNA in melanoma cell lines

Northern blot analysis identified a single band of 4.3 kb in total RNA from a heart sample (20 µg) after exposure for 3 days, similar to the size reported in other human tissues (Hosoda et al. 1991). However, no product was seen in any of the melanoma cell lines [1 µg of poly($A^+$)], even after exposure of the blot for 7 days. Hybridization signals with the G3PDH probe confirmed roughly equal loading of poly(A)+ and total RNA from the melanoma cell lines and heart tissue (data not shown). Absence of ETA mRNA detected by Northern blot suggested that the level of ETA receptor expression was very low. In the melanoma cell lines, Northern blot analysis using 80 µg of total RNA from each of the melanoma lines DX3K, LT5.1 and $HMB_2$ demonstrated a signal at 1.9 kb in all the cell lines studied, after exposure to a high-sensitivity film for 7 days. No signal at the position of 4.3 kb was identified, corresponding to the wild-type ETA (Figure 5).

### Stable transfection of the truncated ETA cDNA and ligand binding

The truncated and wild-type ETA cDNA coding regions were stably transfected into CHO cells, as confirmed by RT-PCR. The amount of bound radioactivity of the cells expressing the truncated ETA mRNA was similar to non-specific binding and showed a linear relationship with the concentration of ET-1, indicating that ET-1 did not bind to the cells. Cells expressing the wild-type ETA showed saturable binding to ET-1. Neither truncated nor wild type ETA-transfected cells bound ET-3 (Figure 6).

### DISCUSSION

A truncated ETA mRNA transcript in which exon 3 and exon 4 have been skipped was identified by RT-PCR and is most probably produced by alternative pre-mRNA splicing. In the constitutive pre-mRNA splicing of ETA receptor genes, all introns are spliced out and eight exons are subsequently connected in order, producing wild-type ETA mRNA. In the truncated ETA transcript, however, exons 3 and 4 are spliced out together with their introns, exon 2 joining directly to exon 5. Previous studies have shown exon 3 and exon 4 encode 109 amino acids, starting from the second amino acid of the first extracellular loop through transmembrane domain III, the second intracellular loop, then through transmembrane domain IV until the amino terminus of the second extracellular loop (Hosoda et al. 1992). The absence of exon 3 and exon 4 will result in deletion of this area from the encoded protein (Figure 7). This transcript has been described previously (Miyamoto et al. 1996). Alternative pre-mRNA has been found in numerous genes and such differential splicing is a very effective way to generate functional diversity from a single gene. Alternative splicing is known to exist in a number of the G-protein-coupled receptor superfamilies, such as dopamine receptors (Giros et al. 1991) and 5-hydroxytryptamine (5-HT) receptors (Canton et al. 1996). Two human ETB variants produced by alternative splicing have been reported (Shyamala et al. 1994; Elshourbagy et al. 1996).

It was shown in this study that the wild-type and truncated ETA are widely expressed in many normal tissues. The dominant receptor RNA is the wild type. Both receptor mRNAs have also been detected in fetal heart tissue as early as 12 weeks. However, in melanoma cell lines and melanoma, the dominant receptor mRNA type detected by RT-PCR is the truncated ETA receptor. Using Northern blot analysis, the wild-type ETA mRNA was not detected, demonstrating that the level of the wild-type ETA expressed was very low in melanoma cell lines studied. The identification of a 1.9-kb band by Northern blot suggests that this signal may be the truncated ETA mRNA.

ETs play an important role in the normal development of the neural crest and of cells of neural crest origin, including melanocytes. Previous studies have shown that ETs can promote the proliferation and differentiation of precursor melanocytes (Reid et al. 1996), and a role for ETs in the biology of melanomas has been suggested by several workers (Yohn et al. 1994; Kikuchi et al. 1996). ET-1 is known to be a weak mitogen but a strong chemoattractant in melanoma cells (Yohn et al. 1994), suggesting the importance of the role of ETs in the metastatic process of melanoma. ETB receptors have been detected by Northern blot analysis, RT-PCR and pharmacological studies in a number of melanoma cell lines. However, ETA mRNA has not been detected by Northern blot analysis (Yohn et al. 1994). A possible reason for this may be that not enough RNA was loaded, as our study showed that the mRNA expression level of ETA in melanoma cells is very low. Kikuchi et al (1996) did not detect ETA in several cell lines from metastatic lesions even using RT-PCR, but this study used primers in exons 3 and 4 for amplification. This explains the discrepancy between our own RT-PCR data in melanoma cell lines

© Cancer Research Campaign 1998

and that of Kikuchi et al, because we have demonstrated exons 3 and 4 are absent in the truncated ETA receptor mRNA.

Structural–functional studies have suggested that the amino region (transmembrane I–III plus intervening loops) of the human ETA receptor is the key region for ETA receptor selectivity, and several amino acids in the boundary of the second transmembrane domain and the first extracellular loop are important for binding affinity for its ligand, ET-1 (Sakamoto et al, 1993; Adachi et al, 1994; Becker et al, 1994). The absence of the region encoded by exons 3 and 4 will result in the lack of part of the functional region. Our binding studies demonstrate that absence of this region resulted in loss of binding affinity for the ligand ET-1.

A previous study showed that split fragments at any intracellular or extracellular loop (except at the first intracellular loop) of a G-protein-coupled rat muscarinic acetylcholine receptor were incorporated into the plasma membrane but the binding affinities for its ligands was lost, suggesting that plasma membrane insertion of G-protein-coupled receptor does not require the presence of a full-length receptor protein (Schoneberg et al, 1995). A truncated form of 5-$HT_{2c}$ receptor produced by alternative splicing did not bind to its ligands, although it was expressed on the cell membrane (Canton et al, 1996). We suggest that the truncated ETA receptor will still target the membrane with five transmembrane domains, two extracellular and two intracellular loops, but different conformation of the receptor may make it lose its binding affinity for its ligand ET-1 and it may bind to some other ligand and play a different role from wild type ETA. It may also interact with wild type ETA/ETB receptors or other proteins/receptors for ETs or other cytokines to modulate their functions, or it may regulate signal transduction pathways.

An alternative outcome, although less likely, is that the truncated ETA may remain in the cytoplasm as a result of the protein's conformational change and, thus, fulfil a new function. Examples of cytoplasmic truncated forms of membrane receptors have been found in other receptor families (Faure et al, 1996).

In conclusion, a truncated human ETA receptor with exons 3 and 4 skipped because of alternative pre-mRNA splicing has been found in a variety of tissues, and the truncated transcript is the dominant .ETA receptor mRNA in melanoma cell lines and melanomas. The absence of the correspondingly encoded amino acid region results in the loss of affinity for its ligand, ET-1. Further studies will be needed to investigate its subcellular location and its roles in cell growth.

### ACKNOWLEDGEMENT

This study was supported by an award from the Sino-British Friendship Scholarship Scheme.

### REFERENCES

Adachi M, Furuichi Y and Miyamoto C (1994) Identification of a ligand-binding site of the human endothelin-A receptor and specific regions required for ligand selectivity. *Eur J Biochem* **220**: 37–43

Arai H, Hori S, Aramori I, Ohkubo H and Nakanishi S (1990) Cloning and expression of a cDNA encoding an endothelin receptor. *Nature* **348**: 730–732

Arai H, Nakao K, Takaya K, Hosoda K, Ogawa Y, Nakanishi S and Imura H (1993) The human endothelin B receptor gene. Structural organisation and chromosomal assignment. *J Biol Chem* **268**: 3463–3470

Bax WA, Bos E and Saxena PR (1993) Heterogeneity of endothelin/sarafotoxin receptors mediating contraction of the human isolated saphenous vein. *Eur J Pharmacol* **239**: 267–268

Becker A, Haendler B, Hechler U and Schleuning WD (1994) Mutational analysis of human endothelin receptor $ET_A$ and $ET_B$. Identification of regions involved in the selectivity for endothelin 3 and cyclo-(D-Trp-D.Asp-Pro-D-Val-Leu). *Eur J Biochem* **221**: 951–958

Canton H, Emeson RB, Barker EL, Backstrom JR, Lu JT, Chang MS and Sanders-Bush E (1996) Identification, molecular cloning, and distribution of a short variant of the 5-hydroxytryptamine$_{2C}$ receptor produced by alternative splicing. *Mol Pharmacol* **50**: 799–807

Chomczynski P and Sacchi N (1987) Single-step method of RNA isolation by acid guanidinum thiocyanate-phenol-chloroform extraction. *Anal Biochem* **162**: 156–159

Elshourbagy NA, Adamou JE, Gagnon AW, Wu HL, Pullen M and Nambi P (1996) Molecular characterization of a novel human endothelin receptor splice variant. *J Biol Chem* **271**: 25300–25307

Faure E, Gouedard L, Imbeaud S, Cate R, Picard JY, Josso N and Clemente N (1996) Mutant isoforms of the anti-mullerian hormone type II receptor are not expressed at the cell membrane. *J Biol Chem* **271**: 30571–30575

Giros B, Martres MP, Pilon C, Sokoloff P and Schwartz JC (1991) Shorter variants of the $D_3$ dopamine receptor produced through various patterns of alternative splicing. *Biochem Biophys Res Commun* **176**: 1584–1592

Hosoda K, Nakao K, Arai H, Suga S, Ogawa Y, Mukoyama M, Shirakami G, Saito Y, Nakanishi S and Imura H (1991) Cloning and expression of human endothelin-1 receptor cDNA. *FEBS Lett* **287**: 23–26

Hosoda K, Nakao K, Tamura N, Arai H, Ogawa Y, Suga S, Nakanishi S and Imura H (1992) Organization, structure, chromosomal assignment, and expression of the gene encoding the human endothelin-A receptor. *J Biol Chem* **267**: 18797–18804

Inoue A, Yanagisawa M, Kimura S, Kasuya Y, Miyauchi T, Goto K and Masaki T (1989) The human endothelin family: three structurally and pharmacologically distinct isopeptides predicted by three separate genes. *Proc Natl Acad Sci USA* **86**: 2863–2867

Kikuchi K, Nakagawa H, Kadono T, Etoh T, Byers HR, Mihm Jr MC and Tamaki K (1996) Decreased $ET_B$ receptor expression in human metastatic melanoma cell. *Biochem Biophys Res Commun* **219**: 734–739

Miller AD and Rosman GJ (1989) Improved retroviral vectors for gene transfer and expression. *Bio Techniques* **7**: 980–988

Miyamoto Y, Yoshimasa T, Arai H, Takaya K, Ogawa Y, Itoh H and Nakao K (1996) Alternative RNA splicing of the human endothelin-A receptor generates multiple transcripts. *Biochem J* **313**: 795–801

Mombouli JV, Le SQ, Wasserstrum N and Vanhoutte PM (1993) Endothelins 1 and 3 and big endothelin-1 contract isolated human placental veins. *J Cardiovasc Pharmacol* **22**: S278–S281

Ogawa Y, Nakao K, Arai H, Nakagawa O, Hosoda K, Suga S, Nakanishi S and Imura H (1991) Molecular cloning of a non-isopeptide-selective human endothelin receptor. *Biochem Biophys Res Commun* **178**: 248–255

Reid K, Turnley AM, Maxwell GD, Kurihara Y, Kurihara H, Bartlett PF and Murphy M (1996) Multiple roles for endothelin in melanocyte development: regulation of progenitor number and stimulation of differentiation. *Development* **122**: 3911–3919

Riezebos J, Watts IS and Vallance PJT (1994) Endothelin receptor mediating functional responses in human small arteries and veins. *Br J Pharmacol* **111**: 609–615

Sakamoto A, Yanagisawa M, Sawamura T, Enoki T, Ohtani T, Sakurai T, Nakao K, Toyo-oka T and Masaki T (1993) Distinct subdomains of human endothelin receptors determine their selectivity to endothelin$_A$-selective antagonists and endothelin$_B$-selective agonists. *J Biol Chem* **268**: 8547–8553

Sakurai T, Yanagisawa M, Takuwa Y, Miyazaki H, Kimura S, Goto K and Masaki T (1990) Cloning of a cDNA encoding a non-isopeptide-selective subtype of the endothelin receptor. *Nature* **348**: 732–735

Schoneberg T, Liu J and Wess J (1995) Plasma membrane localization and functional rescue of truncated forms of a G protein-coupled receptor. *J Biol Chem* **270**: 18000–18006

Shyamala V, Moulthrop THM, Stratton-Thomas J and Tekamp-Olson P (1994) Two distinct human endothelin B receptors generated by alternative splicing from a single gene. *Cell Mol Biol Res* **40**: 285–296

Tso JY, Sun XH, Kao TH, Reece KS and Wu R (1985) Isolation and characterization of rat and human glyceraldehyde-3-phosphate dehydrogenase cDNAs: genomic complexity and molecular evolution of the gene. *Nucleic Acids Res* **13**: 2485–2502

Yohn JJ, Smith C, Stevens T, Hoffman TA, Morelli JG, Hurt DL, Yanagisawa M, Kane MA and Zamora MR (1994) Human melanoma cells express functional endothelin-1 receptors. *Biochem Biophys Res Commun* **201**: 449–457

© Cancer Research Campaign 1998